# Stress fracture of bilateral tibial metaphysis due to ceremonial march training: a case report

**DOI:** 10.1186/1757-1626-3-3

**Published:** 2010-01-04

**Authors:** Mustafa Kurklu, Sener Ozboluk, Erden Kilic, Oner Tatar, Huseyin Ozkan, Mustafa Basbozkurt

**Affiliations:** 1 GATA Department of Orthopedics and Traumatology, Etlik, Ankara, Turkey

## Abstract

Stress fractures are caused by repetitive microtraumas that occur during unusual or increased activities. Clinical suspicion is essential for the diagnosis. A twenty-years old soldier was presented with bilateral knee pain and restriction of knee movements after a period of training for ceremonial march. Although plain X-rays were normal, scintigraphy and MRI revealed stress fractures at metaphyseal region of both tibias. History of a patient presenting with persisting joint or bone pain after an unusual repetitive activity should be delicately inquired. Typical history, although pain might be localized to unusual sites, should raise the suspicion of a stress fracture.

## Background

Stress fractures are caused by repetitive microtraumas that occur during increased or unusual activities. Tibia, metatarsals and calcaneus are the most commonly affected sites. Clinical suspicion is essential for the diagnosis. Although observed in all ages, stress fractures are common in adolescent athletes and recruits [1-5]. Conventional radiography alone may not be sufficient for diagnosis. Scintigraphy and MRI facilitates the diagnosis and increase the number of cases diagnosed [5].

Osteonecrosis, osteochondritis dissecans, ligament injuries, periosteal reactions, osteomyelitis and bone tumors should be considered in the differential diagnosis [6]. Particularly for the stress fractures of tibia differential diagnosis also includes periostitis and musculotendinous injuries [7].

In this report, we presented a case of bilateral tibial plateau stress fracture following training for ceremonial march which is typically performed by violent foot strikes on the ground and the case was discussed the in light of the literature.

## Case Presentation

**Figure 1:**
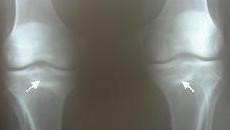
**Anteroposterior radiographs show transverse sclerotic lesions on metaphyseal region of both tibias (arrows) where left one extends into the tibial plateau**.

**Figure 2:**
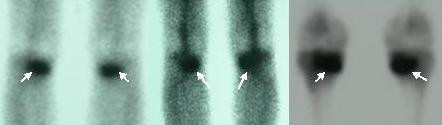
**Oval-shaped increased osteoblastic activity on the metaphyseal region of both tibias (arrows)**.

**Figure 3:**
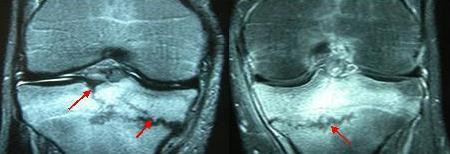
**Coronal MR images show a fracture line at the medial side of left tibia which extends into the lateral half**. Fracture at the right side extends into the articular surface.

The patient was 20 years-old male private, recruited 35 days ago, with no history of previous bone or joint disease. One month before development of symptoms, he started training for ceremonial march which typically involved violent foot strikes on concrete ground and lasted for at least one hour. He experienced right knee pain at 13^th ^day and left knee pain at 15^th ^day of training. Initially (within the first three days) pain was felt only during ceremonial march, which relieved during rest or activities of daily life. Later, pain became persistent during the activities of daily life. His first radiographs of both knees (at the 18^th ^day) showed no pathology. At his physical examination performed at the first month, his left knee was painful and extension was restricted for the last 10 degrees while flexion was restricted for the last 20-25 degrees; his right knee was also painful and extension was restricted for the last 15-20 degrees while flexion was restricted for the last 20 degrees. Medial sides of his both knees were tender on palpation and pain was elicited during varus and valgus stress tests. X-rays of both knees showed transverse sclerotic lesions and cortical thickening at the medial plateaus (Figure-1). Three-phase bone scintigraphy (Tc99m MDP) performed to disclose a stress fracture showed an increased osteoblastic activity of oval-shape at the medial side of tibial metaphysis (Figure-2). MRI showed a fracture line with an intraarticular extension and bone marrow edema that surrounded the fracture (Figure-3).

Patient was diagnosed to have bilateral tibial stress fracture and immobilized in long leg cast at 15 degree flexion for 6 weeks. Non-steroidal anti-inflammatory drug was given for 5 days. Partial weight bearing with crutches was allowed after removal of the cast and full range of flexion and extension was achieved by CPM within 15 days. Passive exercises of quadriceps and hamstring muscles were performed by TENS followed by active exercises at 2^nd ^month. Patient was allowed full-weight bearing at the end of 3^rd ^month. He returned to his military unit at 6^th ^month with prohibition of exercises with axial impaction.

## Conclusion

It is imperative to collectively evaluate the history, physical examination and radiological findings to diagnose a stress fracture. Pain that develops after unusual activities, increases during physical activity and relieves by rest; but persists for a several weeks is typical for a stress fracture. Physical examination presents no other finding except a localized pain. History of our case and pain localized to medial side of both knees were typical of stress fracture.

Walking on hard surfaces increases the axial compressive forces acting on the knee joint. Milgrom et al have determined that axial compressive and tensile forces were 48-285% higher during overground running compared to treadmill [8]. Additionally, McKenzie et al have shown that strike of the foot at slight pronation, as it does during ceremonial march, increases the forces acting on the medial compartment of the knee [9]. We believe that stress fractures of the medial side both knees in our case following ceremonial march were caused by the combination of above-described forces.

Initial x-rays are usually normal in stress fracture [10]. Horizontal radiolucent lines, cortical thickening and periosteal reactions are the most frequent findings that can be subsequently determined. In our case, initial radiographs were normal; however radiographs taken one month later showed the sclerotic radiolucent lines at both tibial metaphysis.

Scintigraphy may give false negative or positive results at the beginning [10]. Fusiform or oval shaped increased activity is typical for stress fracture but a transverse stress fracture may present with decreased activity [10-12]. We have observed a typical oval shaped increased activity by scintigraphy.

Treatment is symptomatic and usually includes conservative treatment with 4 to 6 weeks of immobilization in a long leg cast and NSAIDs, but surgical treatment may be preferred in adolescents and athletes depending on the type of fracture [13,14]. We preferred six weeks of immobilization in a long leg cast and NSAIDs for 5 days.

Wearing inappropriate shoes, repetitive walking or running on hard surfaces for long periods and pre-existing biomechanical deformities increase the risk for stress fracture. Establishing an exercise and training program specific to individual's physical condition and modifications in daily life such as a night sleep of at least 6 hours may decrease the risk for a stress fracture [10,14]. In our case, development of stress fracture at the 13^th ^day of military service may be attributed to overtraining compared to patient's physical condition and to the ceremonial march exercises performed on a hard surface.

As for conclusion, keeping stress fractures in mind, particularly if history is typical and pain is localized to bones with high risk for a stress fracture such as tibia, metatarsals and calcaneus, even initial x-rays may appear normal, will provide early diagnosis and treatment of stress fractures.

## Consent

Written informed consent was obtained from the patient for publication of this case report and accompanying images. A copy of the written consent is available for review by the Editor-in-Chief of this journal.

## Competing interests

The authors declare that they have no competing interests.

## Authors' contributions

MK was in-charge of the patient and conducted the imaging studies.

SO reviewed the literature and contributed to writing of the report.

EK reviewed the literature, contributed to writing of the report and translated to English.

OT helped MK for organization of imaging studies and also treatment and rehabilitation of the patient.

HO contributed to writing of the report.

MB is the chairman of the department, provided consultation for both treatment of the patient and writing of this report.

All authors read and approved the final manuscript.
